# Pathological progress of traumatic femur head necrosis after femoral neck fracture in children and adolescents: a case series study

**DOI:** 10.1093/jhps/hnab025

**Published:** 2021-04-20

**Authors:** Fan Yang, Zhikun Zhuang, Yonggang Tu, Zhinan Hong, Fengxiang Pang, Wei He, Qiushi Wei, Ziqi Li

**Affiliations:** 1 Department of Orthopaedics & Traumatology, Lingnan Medical Research Center, Guangzhou University of Chinese Medicine, No.12, airport road, Baiyun distinct, Guangzhou city, Guangdong province, 510000, China; 2 Department of Joint Diseases, Quanzhou Orthopedic-traumatological Hospital of Fujian Traditional Chinese Medicine University, No.61, Citong western road, Quanzhou city, Fujian province, 362000, China; 3 Department of Orthopaedics, Dongguan Eastern Central Hospital, No. 88, Changdong Road, Changping Town, Dongguan city, Guangdong province, 510000, China; 4 Department of Joint Diseases, Traumatology & Orthopedics Institute of Guangzhou University of Chinese Medicine, The Third Affiliated Hospital of Guangzhou University of Chinese Medicine, Guangzhou city, Guangdong province, 510000, China

## Abstract

The pathological progression and prognosis of traumatic femur head necrosis (TFHN) after femoral neck fracture (FNF) in children and adolescent is relatively unknown and has never been specifically characterized. As we speculated, the prognosis in such population would be poor and characterized as the high risk of femoral head collapse, hip deformity and degeneration in a short term. This retrospective case series enrolled 64 children and adolescent with TFHN who treated with observational treatment from 2000.1 to 2018.1. The primary outcomes, the progression of femoral head collapse, hip deformity (Stulberg classification) and hip degeneration (Tönnis grade), and their prognostic factors were analysed. Sixty-four patients with a mean age of 13 years (6–16 years) were included. A total of 28 hips (44%) showed unsatisfactory outcome and 25 (39%) hips collapsed progressively during a mean follow-up of 48 months (24–203 months). Finally, 38 hips (59%) experienced hip deformity, 20 of them were Class IV/V. Thirty-four hips (53%) generally progressed to osteoarthritis, 14 of them were classified as Grades II/III. The location of the lesion and the presence of subluxation were found to be related to progression of collapse; however, the presence of subluxation was the only independent risk factor of severe hip deformity and degeneration. TFHN in children and adolescent is a rapidly progressing disease with a poor prognosis characterized by a high risk of femoral head collapse progression. If the subluxation emerged, collapsed cases showed increasingly tendency towards hip deformity and degeneration.

## INTRODUCTION

Traumatic femur head necrosis (TFHN) is a potentially disabling complication that occurs after femoral neck fracture (FNF) in children and adolescent [1, 2]. As TFHN is the most common complication, a meta-analysis revealed an average incidence of 23.5% [3]. And, another retrospective study, as the largest known sample size for paediatric FNF, indicated an incidence of 24.5% for TFHN [4]. However, this condition remains unfamiliar to most orthopaedic surgeons because of the rare incidence of primary injury [5, 6]. Numerous studies have attempted to elucidate the incidence of TFHN and the relevant risk factors, but few have provided the characteristics of the pathologic progression, prognosis or the targeted treatment. Mirrored to the experience of adult FHN, or other childhood osteonecrosis caused by other aetiologies, hip-preserving operations, such as core decompression [7, 8], free vascularized-fibular grafting [9] and hip osteotomy [10], have been used without confirmed evidence. In the absence of clear guidance, surgeons failed to make timely treatment plan, and observational treatment, such as non-weight bearing exercises [5, 11] or pharmacotherapy [12], were used reluctantly.

As we have observed in clinical practice, TFHN rarely has a promising prognosis in children and adolescent. These patients were seemingly present with a strong tendency for femoral head collapse, a crucial pathologic change leading to poor outcomes, and hip deformity that occurs with skeletal maturity, which indicates a high risk of hip degeneration in immature patients [13]. We believe that these indicators, including femoral head collapse, hip deformity and degeneration, are the basis of the disease progression of TFHN in children and adolescent if they have not received proper treatment, but that have not yet been described in detail.

To the best of our knowledge, the largest sample size to date of 64 patients was included in the current study. We aimed to analyse the disease progression of TFHN in children and adolescent with a hypothesis that the prognosis in this paediatric population would be poor because of the high risk of femoral head collapse, hip deformity and osteoarthritic change.

## MATERIALS AND METHODS

This study was a retrospective case series study and was reported according to the STROBE statement [14]. After the approval of the Institute Ethics Committee (NO. ZYYECK[2020]053), a retrospective case study was conducted based on patients with TFHN in children and adolescent in our institute from 2000-1 to 2018-1, according to the following inclusion criteria: (i) participants diagnosed with TFHN [1, 15] as a complication of a previous femoral neck fracture; (ii) patients with no history of corticosteroid administration or alcohol abuse; (iii) Epiphyseal plate of femoral head were not closed completely when the fracture occurred; (iv) patients with no other complications from FNF, such as nonunion or infection; (v) patients who completed a follow-up period of more than 2 years; and (vi) patients who had not received hip-preserving surgery after diagnosis (patients received surgery have already appeared severe femoral head deformity or osteoarthritis changes).

The treatment of included patients consisted of pain relief, restricted weight bearing and physical treatment, within the first 2 years after diagnosis of TFHN. We did not use any type of anti-osteoporosis medication because of the unknown complications in children and adolescents. Symptomatic treatments consisted of non-steroidal anti-inflammatory drugs (NSAIDs) or analgesics, as needed. The clinical and radiographic data were assessed at time of TFHN diagnosed and serially every 3–4 months within the first 2 years after treatment and every 6 months in the third year and subsequently.

Unsatisfactory outcomes were characterized by the conversion to THA or the presence of a ‘fair or poor result’ according to Ratliff’s clinical criteria [1]. Good was defined as ‘clinical, no or negligible pain; full or minimal restrictive hip movement; and normal or mild restricted activity’. Fair was defined as ‘clinical occasional pain; hip movement restriction <50%; and normal or mild restricted activity’. Poor was defined as ‘clinical or disabling pain; hip movement restriction >50%; and restricted activity’.

Radiographic data were collected, including X-ray, CT and MRI data. According to the classification system of the Japanese Investigation Committee (JIC) [16], all hips were classified into four types. Types A, B and C1 were assigned to groups where the necrotic area did not extend to the acetabular edge (inside coverage). Type C2 was assigned to groups where the necrotic area expanded beyond the acetabular edge (outside coverage). Then, we analysed whether the location of the necrotic area affected the prognosis. The degree of collapse was also evaluated by concentric circles on both anteroposterior and lateral radiographs referring to previous literatures [17, 18]. With the increasing progression of femoral head collapse, subluxation was defined when Shenton’s line was not continuous, and the inadequate acetabular coverage was measured using the lateral center-edge angle (CE angle). The cases with CE angle <25° indicate inadequate coverage of the femoral head [19, 20]. All radiographic quantitative evaluations were performed using ImageJ (1.52a, National Institutes of Health, Bethesda, MD, USA).

Three primary outcomes were evaluated as follows: (i) the progression of femoral head collapse more than 2 mm, (ii) the Stulberg classification and (iii) the Tönnis grade. The Stulberg classification [13] was used to describe the shape of the femoral head at skeletal maturity and to predict the long-term outcome at the last follow-up. The degree of osteoarthritis was defined according to the Tönnis grade (Grade 0–3) [21]. The Stulberg type IV/V was defined as severe hip deformity and Tönnis grade 2/3 was defined as degeneration change. All the hips in all patients at the last follow-up demonstrated skeletal maturity. All the radiographic characteristics and outcomes were evaluated by two experienced orthopaedic surgeons simultaneously and the consensus was discussed with another third surgeon.

Statistical analyses were carried out to analyse which factors, such as age, the location of the lesion and the presence of subluxation, would affect the primary outcomes. Then, adjusted-ORs were analysed by binary logistic regression. Cox proportional hazard model was used to identify the adjusted-HRs of predictive factors related to femoral head collapse progression. With an endpoint of the ‘progression of femoral head collapse’ according to X-ray images, a Kaplan–Meier analysis was performed, and survival curves were created with the log-rank test. A value of *P* < 0.05 was considered to indicate significance. These statistical analyses were performed using SPSS Statistics (version 25; IBM, New York, NY, USA).

## RESULTS

**Fig. 1.**
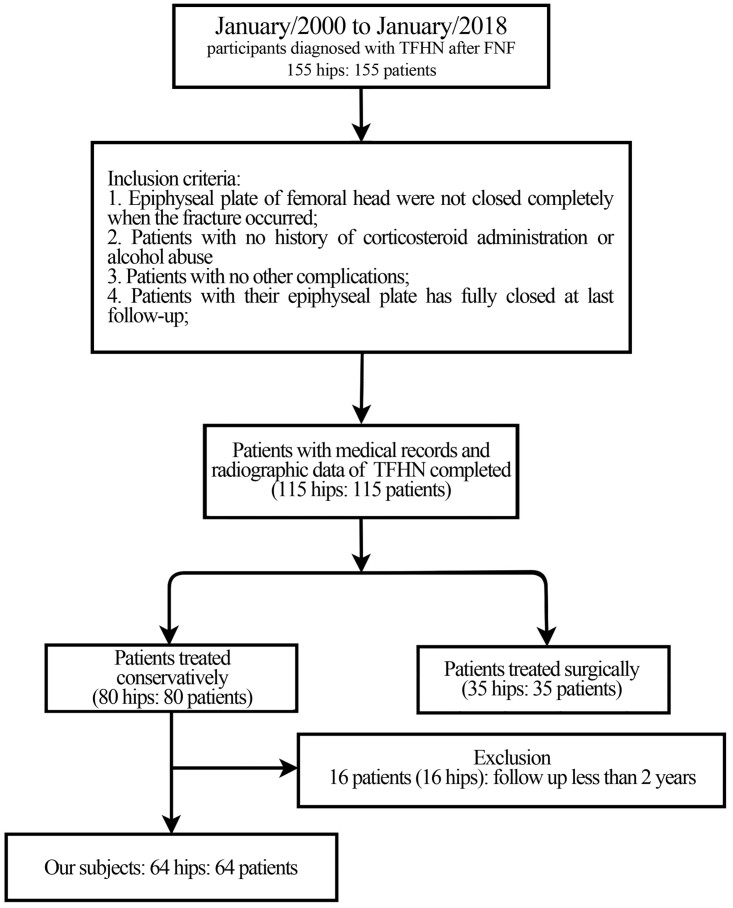
The flow chart of TFHN after FNF in children and adolescent underwent observational treatments.

A total of 155 children and adolescents (155 hips) were diagnosed with TFHN from 2000-1 to 2018-1. After screening, 115 patients (115 hips) have complete medical record and radiograph data; 35 patients (35 hips) treated with surgery were excluded; and 16 patients lost to follow up in 2 years after the diagnosis of TFHN because of some personal reasons as they chose another hospital or lost contact. Sixty-four patients were finally analysed in our studies (Fig. 1). The basic information of patients was shown in Table I. The mean follow-up time was 48.38 months (24–203 months). When patients were diagnosed, 14.1% (9/64) of hips exhibited subluxation and 10.9% (7/64) exhibited inadequate coverage of the femoral head on imaging. Then, 29.7% (19/64) of hips had subluxation after an average of 33.6 months (24–69 months). At the last follow-up, according to Ratliff’s clinical criteria, 36, 17 and 11 patients showed good, fair and poor outcomes, respectively. Five patients with poor clinical outcomes underwent total hip arthroplasty.

**Table I.** The characteristics of TFHN and unadjusted analysis of factors related to primary outcomes

Characteristics	*n*/mean value	P value		
		Collapse progression^*a*^	Severe hip deformity^*b*^	Severe hip degeneration^*b*^
Male/female	42/22	0.014	0.292	0.452
Age (years)	13.28	0.944	0.787	0.211
Internal fixation of FNF (Non-surgery/kirschner wire/hollow screw)	8/37/19	0.224	0.085	0.564
Interval between fracture and TFHN diagnosis (months)	13.08	0.847	0.735	0.972
Symptomatic/asymptomatic	24/40	0.001	0.001	0.001
femoral head collapse or not at time of TFHN diagnosis	33/31	0.010	0.001	0.186
JIC classification (AB/C1/C2)	13/24/27	<0.001	<0.001	0.003
Subluxation/without subluxation	19/45	<0.001	<0.001	<0.001
Adequate/inadequate acetabular coverage	57/7	0.838	0.487	0.651

Abbreviations: FNF, femur neck fracture; JIC, Japanese Investigation Committee; TFHN, traumatic femur head necrosis.

^a^ Cox multivariate regression analysis.

^b^ Binary logistic regression.

**Fig. 2.**
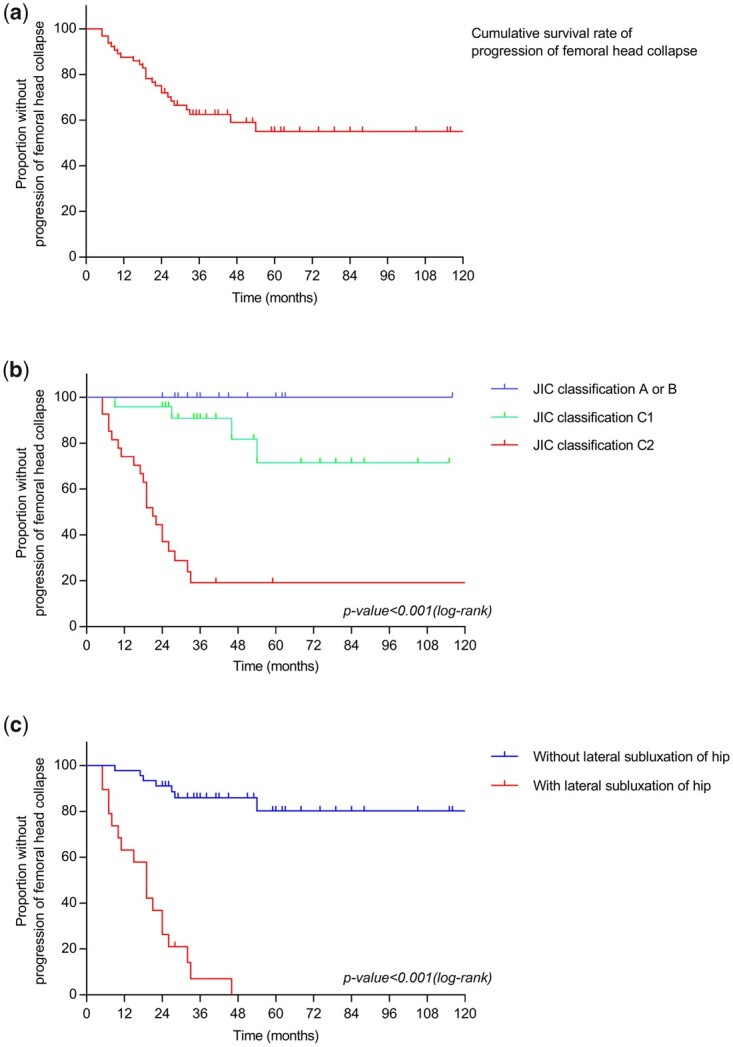
The Kaplan–Meier curve showed the cumulative percentage of the progression of femoral head collapse (**a**) and demonstrated a significant relationship between survival time and prognostic factors (**b**, **c**). With the endpoint of ‘femoral head collapse progressing more than 2 mm’, the overall survival rates were 100.0, 83.3 and 22.2% in the JIC classification A/B, C1 and C2 respectively (b); the overall survival rates were 15.6 and 94.7% in those with and without subluxation of hip during follow-up (c).

Upon TFHN diagnosis, 48.4% (31/64) of hips had collapsed. Throughout the follow-up time, 39.1% (25/64) of hips progressively collapsed by more than 2 mm. And 54.8% (17/31) of the collapsed hips demonstrated collapse progression during follow-up. In addition, 70.3% (45/64) of hips ultimately progressed to the collapsed stage. If we defined collapse progression with an endpoint of more than 2 mm at follow-up, three factors consisted of the appearance of symptoms, JIC classification and subluxation, affected all primary outcomes through univariate analysis. The initial femoral head collapse related to the collapse progression and severe hip deformity (Table I). Furthermore, multivariate analysis indicated that the JIC classification (adjusted-HR = 6.127 95% CI = 1.8–20.9) and the presence of subluxation (adjusted-HR = 5.338, 95% CI = 1.6–17.8) were prognostic factors for the progression of femoral head collapse (Table II). The Kaplan–Meier curve showed the cumulative percentage of the progression of femoral head collapse and demonstrated a significant relationship between survival time and these prognostic factors (Fig. 2).

**Table II.** Adjusted analysis of prognostic factors related to primary outcomes of TFHN in children and adolescent

Relative factors	P value and OR or HR (95% CI)		
	Collapse progression^*a*^	Severe hip deformity^*b*^	Severe hip degeneration^*b*^
Male/female	*P* = 0.897, HR = 0.941 (0.377∼2.350)	—	—
Femoral head collapse (no- collapse vs collapse)	*P* = 0.169, HR = 1.981 (0.748–5.244)	*P* = 0.091, OR = 6.525 (0.741–55.703)	—
Symptom (symptomatic vs asymptomatic)	*P* = 0.410, HR = 0.648 (0.231–1.820)	*P* = 0.847, OR = 0.787 (0.069–8.955)	*P* = 0.324, OR = 2.498 (0.405–15.396)
JIC classification (AB/C1/C2)	*P* = 0.004, HR = 6.127 (1.800–20.853)	*P* = 0.065, OR = 8.764 (0.876–87.696)	*P* = 0.542, OR = 1.825 (0.264–12.617)
Subluxation (subluxation vs without subluxation)	*P* = 0.006, HR = 5.338 (1.604–17.758)	*P* = 0.012, OR = 25.821 (2.064–323.066)	*P* = 0.033, OR = 13.459 (1.235–146.64)

Abbreviations: JIC, Japanese Investigation Committee; TFHN, traumatic femur head necrosis.

^a^ Cox multivariate regression analysis.

^b^ Binary logistic regression.

At the latest follow-up time, 59.4% (38/64) of the patients experienced hip deformity, indicating a devastating result for children and adolescents because of their high demand for hip function for their work and daily life. Specifically, 26 (40.6%), 18 (28.1%) and 20 (31.3%) patients were classified as having Class I/II, III and IV/V hips, respectively, according to the Stulberg classification. In univariate analysis, the type of symptom, JIC classification, initial femoral head collapse and subluxation were four factors related to severe deformity (Table I). However, multivariate analysis indicated that subluxation (adjusted-OR = 25.82 95% CI = 2.1–323.1) was the only indicator of severe hip deformity (Table II).

In terms of the Tönnis grade, 53.1% (34/64) of hips generally progressed to osteoarthritis. Twenty (27.8%) hips were classified as Grade I, 10 (13.9%) and 4 (5.6%) hips were classified as Grades II and III, respectively. In univariate analysis, the type of symptom, JIC classification, initial degree of femoral head collapse and subluxation were four factors that affected the results (*P* < 0.05; Table I). However, multivariate analysis indicated that subluxation of the hip (adjusted-OR = 13.46, 95% CI = 1.2–146.6) was the only indicator of severe hip degeneration.

## DISCUSSION

A series of studies, first reported by Ratliff and his colleagues in the 1960s, have portrayed TFHN as a severe complication secondary to paediatric FNF [1, 2]. Since then, the unsatisfactory outcomes of this condition have been repeatedly reported by numerous studies; however, the specific pathologic progression, prognosis and relevant risk factors for TFHN in children and adolescent remain unknown. This study is the first to address these deficiencies through a retrospective study that included 64 conservatively treated cases, which is thought to be the largest currently utilized sample size. The pathologic progression and prognosis of TFHN in children and adolescent were characterized in detail by femoral head collapse, hip deformity and hip degeneration. The most important finding in the recent study is that TFHN in children and adolescent is a rapidly progressing disease characterized by a high risk of femoral head collapse, deformity and osteoarthritis change. The subluxation of hip was considered to be the most significant risk factor to the poor prognosis of TFHN.

Femur head necrosis in the paediatric population can be induced by both non-traumatic and traumatic aetiologies. The non-traumatic aetiologies include corticosteroid-associated osteonecrosis, Legg–Calve–Perthes disease and the like [22]. Unlike non-traumatic cases, TFHN are far less common due to the rare incidence of FNF in children and adolescents. Thus, therapists might remain unfamiliar with TFHN because of the lack of guidelines and consensus documents. According to our study, a distinct difference exists in the pathologic progression and prognosis between TFHN and other non-traumatic cases of osteonecrosis.

The most intuitive characteristic of TFHN in children and adolescents was the high incidence of femoral head collapse progression, which is generally considered a turning point indicating poor prognosis in the short term [15]. The presence of collapse primarily depended on the lesion size and the location of the necrotic area, as the large involvement of necrotic lesions results in a high risk of femoral head collapse [15, 16]. We believe that patients with TFHN were susceptible to extensive necrosis due to severe damage of blood supply induced by high-energy primary trauma. Ratliff and his team demonstrated the highest incidence of TFHN occupying the total head (Type I), followed by partial necrosis of the epiphysis (type II) and necrosis between the epiphyseal plate and the fracture line (Type III) [1]. A high risk of total head necrosis, ranging from 35.7% to 80.7% [1, 5, 23–26], has been confirmed repeatedly by studies on the same subject. The high risk of large lesions and disease progression does not seem to be common in corticosteroid-associated children and adolescents.

Legg–Calve–Perthes disease is also a common aetiology of childhood osteonecrosis, usually with extensive and severe involvement of the epiphysis. Canavese *et al*. described the prognosis of Legg–Calve–Perthes disease in patients under 6 years old [27]. According to the Catterall classification, 50 hips had mild involvement (Grade I/II), and 116 hips had severe involvement (Grade III/IV). Similarly, 358 patients with Legg–Calve–Perthes disease (mean age, 5.8 years) were followed for more than 5 years with radiographic data by Wiig *et al*. and 87.7% (314/358) of whom exhibited severe involvement (Grade III/IV) [28].

However, these severely involved patients with Legg–Calve–Perthes disease had a better prognosis than TFHN. First, TFHN tended to be associated with severe deformity. The percentages of Stulberg class III and IV/V hips in our study were 28.1% (18/64) and 31.3% (20/64) in patients with TFHN. The matched data for patients with Legg–Calve–Perthes disease were 22.4% (26/116) and 10.3% (12/116) in the study of Canavese *et al*. [27] and 36.6% (115/314) and 19.2% (60/314) in the study of Wiig *et al*. [28]. Second, during our follow-up of children and adolescent with TFHN, hip degeneration progressed rapidly, which was obviously different from patients with Legg–Calve–Perthes disease, which hardly experienced any osteoarthritic changes in the short term until their 40 s and 50 s [13].

In general, TFHN seemed to be more susceptible to hip deformity and degeneration compared with Legg–Calve–Perthes disease in paediatric population. As we speculated, the weakened abilities of bone repair and remodelling with older age were other potential causes of poor prognosis in patients with TFHN. The average age of the included patients in our study was nearly 13 years old. In this age group, patients with bone necrosis lack satisfactory repair and remodelling abilities and show completely different prognosis and pathological processes than younger patients [29, 30]. Spontaneous femoral head repair and remodelling into a spherical shape were never observed in our patients at the collapsed stage. Even with the largest sample size of such condition, the samples still did not report the results for all ages of children and adolescents. For instance, we only included a small proportion of children younger than 11 years of age (six cases), who would presumably have a better prognosis.

Hip incongruency and instability secondary to irreversible deformity were the major causes of rapid cartilage damage. According to our multivariate analysis, as subluxation occurred during the follow-up, the risk increased sharply by 26-fold for severe hip deformity and 13-fold for severe hip degeneration (Table II). Osteoarthritic changes represented the primary cause of arthroplasty in our patients. Our work determined that the pathologic progression and prognosis of TFHN in children and adolescent was specific and different from corticosteroid-associated osteonecrosis or Perthes disease.

**Fig. 3.**
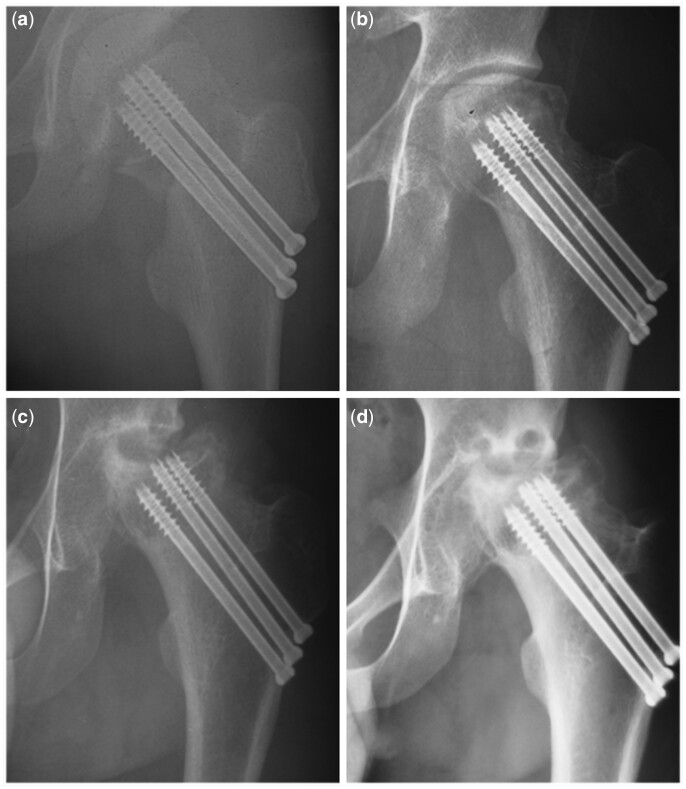
Adolescent of TFHN at collapsed stage showed an increasingly progression towards severe hip deformity and degeneration in the short term, if the subluxation emerged. A male patient of 16 years old, with left FNF, treated with three cannulated screws (**a**). TFHN (JIC classification C2) was diagnosed 1 year after FNF, which already progressed to collapsed stage with subluxation (**b**). During observational treatment, radiographs showed progressively femoral head collapse and subluxation 11 months after diagnosis of avascular necrosis (**c**). At latest follow-up, 2 years after FNF, left hip showed poor outcome according to Ratliff’s clinical criteria, with severe hip deformity (Stulberg classification IV) and hip degeneration (Tönnis grade III).

**Fig. 4.**
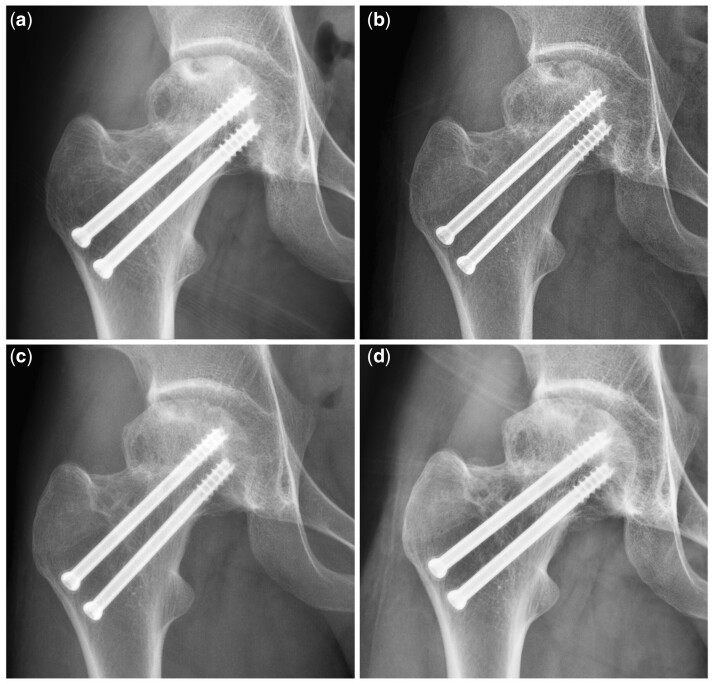
Children with TFHN at collapsed stage showed a satisfactory outcome in a long-term follow-up when their hips were in a stable condition (without subluxation). A male patient of 14 years old, with right FNF, treated with Kirschner wires. TFHN (JIC classification C2) was diagnosed 1 year after FNF (**a**). During observational treatment, radiographs showed progression of femoral head collapse after 1 year follow up, but still without subluxation (**b**). At 2 years follow-up, it still showed no significant progression of collapse and deformity (**c**). After 5 years, the patient still got good outcome according to Ratliff’s clinical criteria, with mild hip deformity (Stulberg classification II) and mild hip degeneration (Tönnis grade I).

As a sign of a ‘head at risk’, subluxation of the hip was defined as the strongest independent risk factor for primary outcomes in our results. For immature patients, subluxation signifies instability of the hip joint. The former is a widely recognized adverse effect of femoral head remodelling, and the latter increasingly exacerbates hip degeneration. Other recognized prognostic factors of progression of femoral head collapse for most types of osteonecrosis of femoral head, such as the initial degree of femoral head collapse [31], JIC classification [16] and symptoms [32], show no apparent relation to severe hip deformity and degeneration. Therefore, these factors did have value for predicting femoral head collapse, but after collapse, not all hip showed further progression to severe hip deformity and degeneration. If the subluxation emerged, cases at collapsed stage showed increasingly tendency towards severe hip deformity and degeneration in short term (Fig. 3), otherwise they might keep satisfactory hip condition (Fig. 4).

However, several limitations still exist. First, the hospitalized patients included in current study have potential risk of selective bias. However, due to the rareness of this kind of disease, the data collection and follow-up of outpatients have much difficulty. Second, we set up a minimum follow-up of 2 years, and finally presented a middle-term result of 4 years on average. To be sure, it was not a long enough period to describe the complete course for most hip problems in paediatric population. However, under the limited follow-up, our results provided a cautionary and useful note of TFHN in children and adolescent, ‘rapid deterioration and relevant factors’, that would be beneficial for clinical decision. Further prospective multi-centre control trials with more cases or control groups are suggested to confirm the results. At last, due to the lack of radiograph data when the fracture happened, we did not analyse the correlations between the prognosis of TFHN and other factors such as initial displacement of the fracture, Delbet classification, time to treatment and residual displacement. And, it would be further studied in future.

In summary, our recent study first identified the characterization of the prognosis of TFHN in child and adolescent. Over half of our patients had hip involvement with osteoarthritic changes or femoral head deformities. Therefore, we believe that TFHN in children and adolescent is a rapidly progressing disease with a prognosis characterized by a high risk of femoral head collapse. For patients who also have hip subluxation, a sign of ‘head at risk’, severe hip deformity and degeneration will occur and result in poor outcomes in the short term. For such conditions, the ideal treatment should be targeted at decreasing the risk of femoral head collapse and rectifying the hip instability induced by subluxation at the same time.
